# Annotations

**Published:** 1896-04-25

**Authors:** 


### Annotations.

### A New Danger for Hospitals.

Hospital authorities will do well to study the case of Alfred Keegan's claim against the Birmingham City Fever Hospital, which was recently decided against the hospital authorities in the County Court. On August 24th one of Keegan's boys was attacked by scarlet fever. The boy was removed to the City Hospital, and there treated until October 8th, when he was sent home. That is, he was kept in hospital for six weeks and three days, rather longer than the usual time. As the boy was being taken home by his parents they noticed a discharging sore behind his ear. Next day Dr. Simpson was called in, and ordered him to be isolated—considering that he was still in an infectious condition. In the result a second child was attacked by the fever, and died. Then another and another became victims of the disease. When all the illnesses had come to an end, Mr. Keegan considered that the City Hospital had actually sent home a patient whilst still in an infectious condition, and that all the suffering and loss to his family had been the result of the defective judgment of the hospital authorities. Accordingly he instituted an action for damages in the County Court, and won it, the judge awarding him fifty pounds. The point to be considered by hospital authorities is, that there are no means known to science whereby we can say with definite precision at what period the infectiousness of a particular scarlet fever case ceases. Medicine is not, like mathematics, an exact science, and, probably, never can be made so.

### The Burial of the Dead.

There can be no doubt that notwithstanding the sanitary advantages of cremation its practice is checked by the horror of it. To a few who are dominated by sanitary ideals; to some whose notions of propriety are outraged by thoughts of decomposition and offensiveness as applied to their own persons; and to others who recoil from the imprisonment of the solid coffin, the brick-lined grave, and the ponderous monument, cremation will be acceptable as a quick and cleanly method of dissolution into ether; but in nothing are nations so conservative as in their modes of burial, and the masses will certainly be slow in accepting the fiery furnace as a proper substitute for laying their dead at rest. A good deal of the difficulty which surrounds the subject at the present time arises from the fact that it is necessary to comply with certain regulations, which appear to have been framed under a complete misconception of the objects of burial. So long as bodies are entombed with the idea of preserving them nuisance must result if the process fails, and ground must be wasted if it succeeds. According to two papers recently read at the Chapter House, St. Paul's Churchyard, by Sir Seymour Haden and Dr. Poore, the difficulty is solved by shallow burial, the very thing that is prohibited by the regulations of the Government. Sir Seymour Haden stated that for the last ten years he had been burying animals, large and small, in the park attached to his house, and digging them up for examination at intervals of from one to five years. He found that, when buried at the regulation depth of four and a-half feet, three to four years were necessary for complete resolution, while if buried at the depth of only a foot one year and a half was sufficient; and if not buried at all, but simply laid on the ground and covered with a foot of earth, the body had completely disappeared, except the bones, in the course of a year, and the mound of earth had been sufficient to prevent all smell. There need be no fear in regard to the amount of ground which would require to be allotted for burial purposes, as an acre of land would prove ample area for a burial ground in perpetuity for a population of 10,000 persons. These things have been said before, but action is restricted, and it would seem at least reasonable that the Government should give as complete facilities for trying a scientific system of burial as it has done for experimenting with the much more expensive process of cremation.

### Sidmouth as a Health Resort.

We have received the annual report of the medical officer of health for Sidmouth, from which it is not difficult to see how pleasant a place it is, and how useful it must be in the as a place of refuge from the cold of winter for those who suffer from many forms of disease which are dangerous in our changeable climate. From the statistics given we may come to the conclusion that Sidmouth is a healthy town, and that it suits very well the constitutions of its inhabitants. We do not think, however, that local statistics are always a fair test of the utility of a place as a health resort, and knowing, as we do, the charms of the place, its picturesqueness, its bright sunshine, the protection it affords from wintry winds when down in the hollow, and the breezy moors which are accessible to those who mount the heights, we nevertheless think that the authorities are well advised in their endeavour to improve its general means of sanitation. The great improvements which have taken place of late years in the water supply, which is now brought from the hills six miles away, obviously necessitates a thorough overhauling of the drainage, and it is a matter for congratulation to find that it is proposed forthwith to take this in hand. It seems to be proposed by the Council, on the recommendation of Mr. Mansergh, the well-known engineer, to relay a portion of the main sewers where they are found to be defective, to remove the present dilapidated and unsightly outfall to the other side of the cliff, out of sight of the town, and to establish a storage tank so as to enable the sewage to be discharged at such a part of the tide as shall prevent the possibility of its being washed back on to the beach in from off the town, and although we gather from the report of the medical officer of health that he is personally opposed to the carrying out of some of these plans, especially the provisions of the tank and the relaying of the main sewer, we cannot but think that if the town wishes to attract visitors, especially visitors who will stay through the winter for the sake of the undoubted advantages of its mild winter climate, its Council will do well to carry out the plan which they have taken in hand.

